# Assessing the Utility of Broad-Acting Inhibitors as Therapeutics in Diverse Venoms

**DOI:** 10.3390/toxins17040188

**Published:** 2025-04-08

**Authors:** Raechel Kadler, Breanna Morrison, Angel Anne Yanagihara

**Affiliations:** 1 Department of Tropical Medicine, Medical Microbiology, and Pharmacology, John A. Burns School of Medicine, University of Hawai’i at Mānoa, Honolulu, HI 96822, USA; rkadler@hawaii.edu; 2 Department of Public Health, University of Birmingham, Birmingham B15 2TT, UK; bmorrison9019@gmail.com; 3 Pacific Biosciences Research Center (PBRC), School of Ocean and Earth Science and Technology, University of Hawai’i at Mānoa, Honolulu, HI 96822, USA

**Keywords:** cubozoa, phospholipase, metalloproteinase, small-molecule inhibitors, therapeutics

## Abstract

Examination of venom constituent bioactivities from diverse venomous animals shows certain highly conserved classes, including enzymes (e.g., phospholipases and metalloproteinases) and pore-forming proteins. While antivenoms targeting other unique and lethal venom components have proven to be life-saving, venom-enzyme-driven tissue damage and morbidity persists. Broad-acting enzyme inhibitors demonstrate the potential to augment antivenom approaches. In this study, we investigate the potential utility of certain broad-acting inhibitors in cubozoa for the first time. Fluorogenic assays were used to determine the phospholipase A_2_ (PLA_2_) and matrix metalloproteinase (MMP) activity of the Hawaiian box jellyfish, *Alatina alata*, and this was compared to representative elapid, viper, and bee venoms. In vitro, evaluation of selected small-molecule inhibitors demonstrated the ability and feasibility of the broad-acting therapeutic doxycycline, which inhibited the PLA_2_ and MMP activity of *A. alata* (approximately 50% reduction at 0.1 mM (95% CI 0.06–0.15) and 2.1 mM (95% CI 1.4–3.0), respectively), in addition to both snake venoms. Additionally, copper gluconate broadly inhibited the PLA_2_ activity of bee, snake, and jellyfish venoms. While all venoms are complex mixtures of bioactive molecules, these studies demonstrate that targeting common class components with broad-acting inhibitors shows promise in clinical and preclinical management.

## 1. Introduction

Envenomations represent a significant public health threat, particularly in remote regions of the tropics and sub-tropics, where access to medical care and resources are limited [1,2,3,4,5,6,7,8]. Jellyfish can be found in aquatic environments across the globe, with species-specific habitats, reproductive behavior, and food source requirements [9]. Their distribution is predicted to expand due to ecosystem disruptions, increasing the associated human health risks [10]. The most clinically relevant class of cnidaria are cubozoans, or box jellyfish [11,12]. This class comprises two orders. The first is Carybdeida, with one to three tentacles per corner of its cuboidal bell. Chirodropida, such as *Chironex fleckeri* (or the “Australian box jellyfish”), have growth-dependent numbers of tentacles, with up to 15 tentacles per corner, each reaching lengths of 3 m [13]. Based on the very limited cases and media outlet reports, Chirodropids have caused at least 35–44 deaths in the Indo-Pacific in the past 24 years [3,4,5,6,7,14,15,16,17,18,19,20,21,22,23,24,25,26,27,28,29,30,31,32]. The actual incidence is much higher, as no formal reporting or surveillance occurs in the most heavily impacted regions [33].

While their ecology and morphology may differ, all cnidarians share the same specialized apparatus for venom delivery: penetrant cnidae or nematocysts [13,34]. Upon contact with prey, or human victims, nematocysts rapidly evert a venom-filled hollow tubule to deliver a complex cocktail of digestive enzymes, proteins, lipids, and small molecules [35,36,37]. Highly conserved classes of venom enzymes incapacitate and digest prey, or play roles in host defense [38]. The most common classes include pore-forming proteins, phospholipases, and matrix metalloproteinases (MMPs) [38]. When injected into human victims, their activity can lead to life-threatening sequelae.

Pore-forming proteins can range in size and permeability but share a conserved mechanism of polymerization and membrane insertion [39], and have been identified in every cubozoan investigated [40,41]. Phospholipases are lipolytic enzymes that hydrolyze membrane lipids, activating the arachidonic acid pathways as well as producing platelet activating factor, causing massive inflammation [42,43,44]. The potential downstream pharmacological effects of phospholipases include neurotoxicity, myotoxicity, cardiotoxicity, anticoagulant effects, platelet aggregation initiation or inhibition, hemolytic activity, and internal hemorrhage [42,43,44]. MMPs degrade basement membranes, the extracellular matrix structures that surround capillary vessels [45,46]. These matrix-degrading enzymes can cause capillary leak, hypotension and shock, severe tissue damage, hemorrhage, and necrosis [47,48,49].

These conserved venom components are also found in diverse phyla including hymenoptera (bees, wasps, and ants), reptiles, and arachnids [43,50,51,52,53,54,55,56] and are associated with tissue damage and morbidity [40,44,45,47,48]. For these reasons, they represent optimal targets for therapeutics [57]. Recently, small-molecule inhibitors of these components have been the focus for treatments of both snakebite and jellyfish envenomations [42,57,58,59,60,61,62]. While antivenoms have been the standard of care for envenomation for over 100 years, they have several limitations. Unfortunately, the availability of antivenom is often limited in the rural and indigenous communities facing the highest risk of envenomations [63,64,65]. Further, antivenoms do not address the tissue-damaging enzymatic components of venom, which can lead to permanent disability [66]. Small-molecule inhibitors could provide an additional modality of treatment with nascent improved accessibility and affordability, and thus improve victim outcomes.

Currently, a small-molecule inhibitor of phospholipase A_2_ (PLA_2_) is in a phase II clinical trial for snakebite treatment [67]. Oral varespladib-methyl is a synthetic indole originally developed to inhibit inflammatory secretory PLA_2_ (sPLA_2_), including type II secreted PLA_2_ [68]. For those who received treatment within 5 h of snakebite, there were improved outcomes based on a composite Snakebite Severity Score (SSS) [69]. Marimastat, which previously passed into Phase III clinical trials as an anti-cancer drug [70], inhibits metalloproteinase through two mechanisms: via its zinc-binding motif and by binding at the enzyme active site [57]. It has demonstrated preclinical efficacy in vivo against haemotoxic snake venoms [60,61,71]. Other small-molecule inhibitors that have been shown to have efficacy against snake venoms include *N*-acetyl-L-cysteine, sodium aurothiomalate, and doxycycline. *N*-acetyl-L-cysteine and sodium aurothiomalate reduced edema, hemorrhage, and myonecrosis following snake venom injection in rat and mouse models, respectively [72,73,74]. Finally, doxycycline interacts with the metal ions necessary for MMP activity, inhibiting their function [75,76]. These therapeutics have the potential to inhibit a broader scope of species by targeting the conserved venom components.

Investigations into the broad-acting capabilities of small molecules against jellyfish have focused on scyphozoan and anthozoan venoms [77,78,79]. Varespladib has been evaluated for its inhibitory capability in *Nemopilemi nomurai* jellyfish; it reduced both phospholipase and hemolytic activity in chromogenic assays [77]. Batimastat is a peptidomimetic hydroxamate similar to marimastat, which was also effective against *N. nomurai* venom activity [77]. Additionally, it inhibited *Cyanea capillata* venom-induced hemorrhagic injury in rats [78]. Development of cubozoan sting treatments have focused on the fast-acting and lethal pore-forming protein component of the venom. Copper gluconate is a patented and potent porin inhibitor, demonstrating efficacy in both in vitro and in vivo models [62,80,81].

This study investigates the ability of selected small molecules to broadly inhibit the venom activity of different animal phyla across multiple enzyme classes. Specifically, we investigated the effects of known inhibitors of snake venom PLA_2_ and MMPs on the venom activities of a representative cubozoan species, *Alatina alata*.

## 2. Results

### 2.1. Phospholipase A_2_ Activity of Snake, Bee, and Jellyfish Venoms

**Figure 1:**
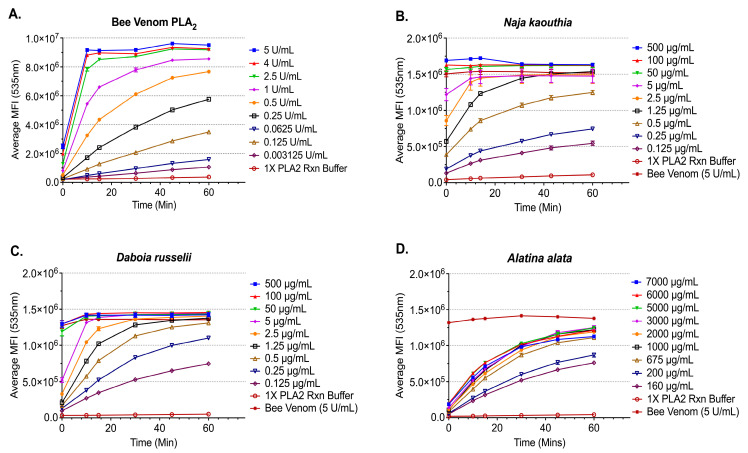
Time- and concentration-dependent phospholipase A_2_ activity. Mean fluorescence intensity (MFI) measurements of PLA_2_ activity from clinically relevant species. (**A**) Honeybee PLA_2_; (**B**) representative elapid *Naja kaouthia*; (**C**) viper *Daboia russelii*; and (**D**) box jellyfish *Alatina alata.* Each point represents the mean of five measurements and the error bars represent standard error of the mean (SEM).

Phospholipase A_2_ activity was determined for representative elapid and viper snake venoms, *N. kaouthia* and *D. russelii*, respectively, as well as a representative box jellyfish *A. alata* (Figure 1). Bee venom PLA_2_ was used to provide a standard curve and serve as a positive control. Each venom demonstrated time- and concentration-dependent PLA_2_ activity.

### 2.2. Inhibition of Phospholipase A_2_ Activities

**Figure 2:**
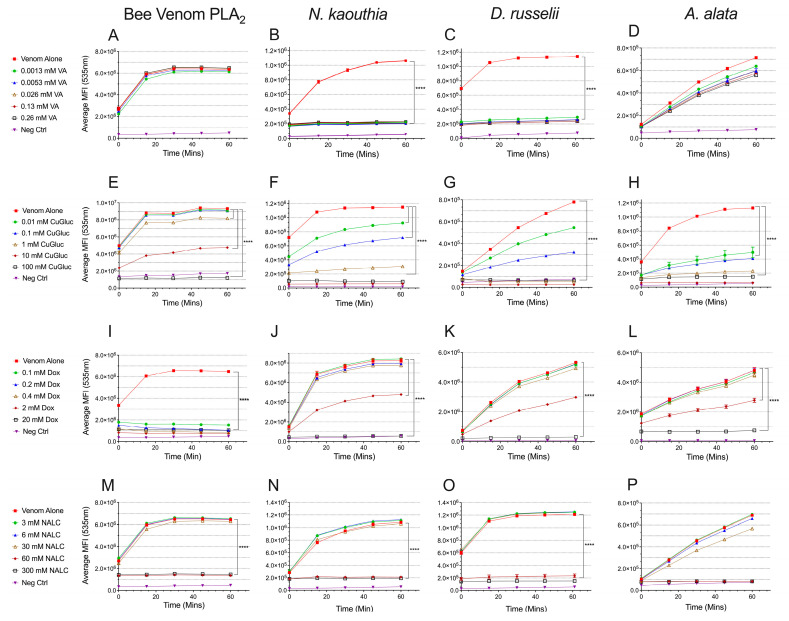
Inhibition of PLA_2_ activity. Inhibition of bee venom PLA_2_ (0.4 U/mL), *N. kaouthia* (0.4 µg/mL or 2 µg/mL), *D. russelii* (200 µg/mL), or *A. alata* (200 µg/mL) venom by (**A**–**D**) VA, varespladib, (**E**–**H**) Dox, doxycycline, (**I**–**L**) CuGluc, copper gluconate, and (**M**–**P**) NALC, *N*-acetyl-L-cysteine. Each curve represents the mean of three measurements and the error bars represent SEM. A 2-way ANOVA (mixed model) compared treatments and time points; a multiple comparisons Dunnett test was used to determine the significance between the venom alone and each treatment. Only *p*-values **** < 0.0001 that were significant across all time points are shown. For a complete list of *p*-values, see Supplementary Table S1. MFI = mean fluorescence intensity.

Selected small molecules were serially diluted in the presence of representative venoms from snake, jellyfish, and hymenoptera to examine their cross-reactive abilities (Figure 2). A two-way mixed ANOVA model with Dunnett’s pairwise comparison tests for significance effects was performed to compare the inhibitors to venom alone over time. Bee venom PLA_2_ activity was inhibited by doxycycline (0.1–20 mM) (*p* < 0.0001), copper gluconate (1–100 mM) (*p* < 0.0001), and *N*-acetyl-L-cysteine (60–300 mM) (*p* < 0.0001) across all time points evaluated (Figure 2E,I,M). Apart from two of the earlier time points (0 min, *p* = 0.0014 and 15 min, *p* = 0.0023, Table S1), varespladib did not reduce bee venom PLA_2_ activity (Figure 2A). However, varespladib completely inhibited both snake venoms at the lowest concentration used, 0.0013 mM (*p* < 0.0001), throughout the 60 min time-course (Figure 2B,C). Doxycycline was also effective at inhibiting the PLA_2_ activity of both snake venoms, although more potently for *N. kaouthia* (Figure 2J,K). *N*-acetyl-L-cysteine significantly (*p* < 0.0001) inhibited both *N. kaouthia* and *D. russelii* venoms at 60 mM and 300 mM, but not at lower concentrations. Copper gluconate, at 10 mM and 100 mM, reduced PLA_2_ activity by approximately 50% and 100%, respectively, throughout the incubation period in both snake venoms evaluated (Figure 2F,G). Similarly, doxycycline (0.1–20 mM) (*p* < 0.0001) and copper gluconate (10–100 mM) (*p* < 0.0001) reduced *A. alata* PLA_2_ activity throughout the time-course (Figure 2H,L). Interestingly, varespladib was not as effective of an inhibitor for the cubozoan venom as compared to the snake venom’s PLA_2_ (Figure 2D).

**Figure 3:**
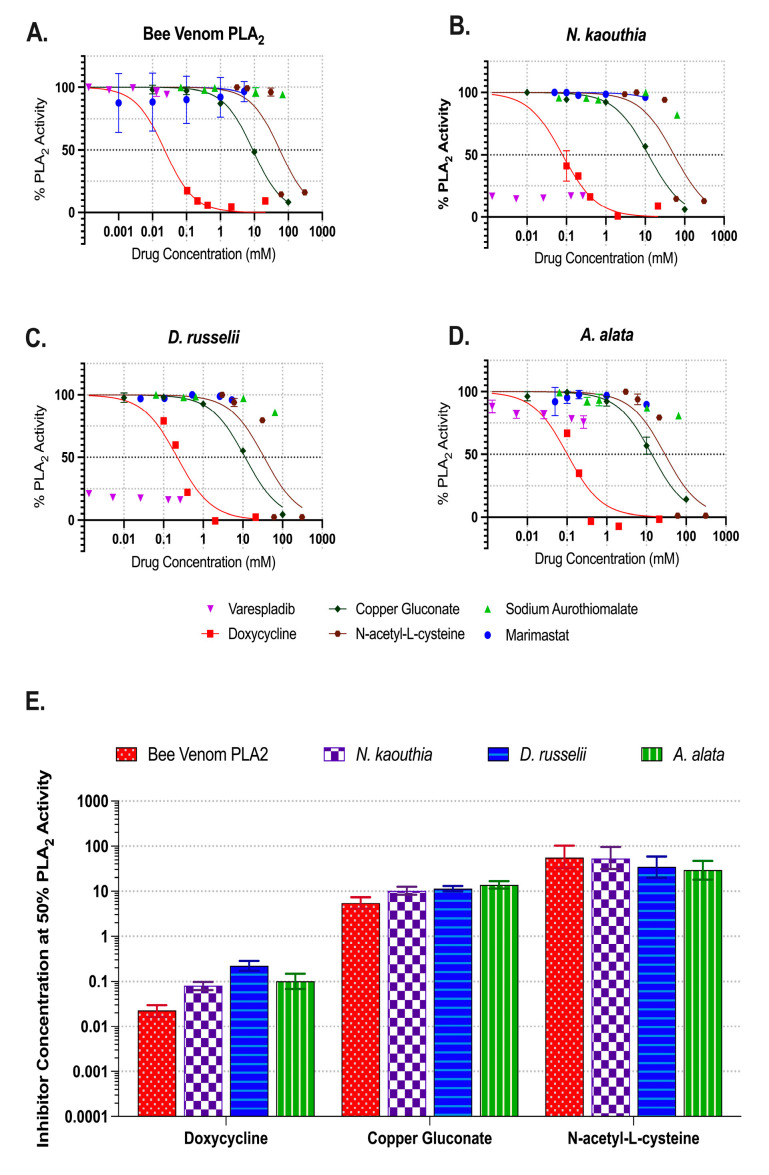
Summary of phospholipase A_2_ activity inhibition by small-molecule drugs. The % PLA_2_ activity was determined for selected inhibitors at 60 min in the presence of (**A**) bee venom PLA_2_ (0.4 U/mL), (**B**) *N. kaouthia* (0.4 ug/mL), (**C**) *D. russelii* (200 ug/mL), and (**D**) *A. alata* (200 ug/mL). Each point represents the mean of three replicates and the error bars represent SEM. (**E**) The inhibitor concentration required to reduce venom PLA_2_ activity by 50% was determined for doxycycline, copper gluconate, and *n*-acetyl-l-cysteine. Bar graphs represent the best fit inhibitor concentration (mM) value for a 50% reduction in PLA_2_ activity and error bars represent 95% confidence intervals.

PLA_2_ activities at the 60 min time point (taken from Figure 2) were normalized and then plotted as a function of inhibitor concentrations (Figure 3A–D). Best-fit values fitting a non-linear regression curve (GraphPad Prism 10) were used to estimate the inhibitor concentration required to yield 50% inhibition (Figure 3E). Varespladib completely inhibited the snake venoms’ PLA_2_ activity (Figure 3B,C) over the selected range, yet was ineffective against bee (Figure 3A) or *A. alata* (Figure 3D) venoms. The concentration required to reduce the venom activity by 50% was either below (snake) or above (jellyfish and bee) the selected range. Doxycycline was broad-acting, requiring less than 0.1 mM for bee venom to 0.2 mM (95% CI 0.17 to 0.29 mM) for *D. russelii* to reduce PLA_2_ activity by 50%. Copper gluconate reduced PLA_2_ activity by 50% for each venom at concentrations between 9.0 mM (95% CI 8.0 to 10.2 mM) for bee venom and 14 mM (95% CI 11.5 to 16.8 mM) for *A. alata*. The concentration of *N*-acetyl-L-cysteine required for a 50% reduction in activity ranged from 29.3 mM (95% CI 18.2 to 47.6 mM) for *A. alata* to 56.4 mM (95% CI 32 to 103 mM) for bee venom. Sodium aurothiomalate and Marimastat were not effective inhibitors of PLA_2_ activity in any venom evaluated (Figure S1A–H).

### 2.3. Gelatinase Activity of Snake and Jellyfish Venoms

**Figure 4:**
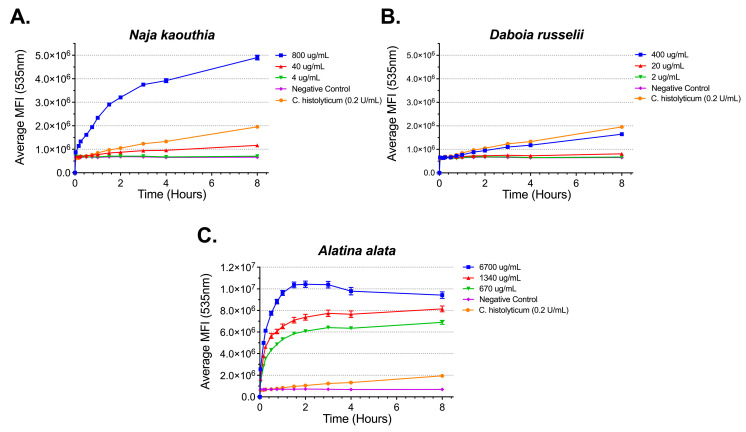
Time- and concentration-dependent gelatinase activity. Mean fluorescence intensity (MFI) measurements of gelatinase activity from (**A**) *N. kaouthia*; (**B**) *D. russelii;* and (**C**) *A. alata*. Each point represents the mean of six measurements and the error bars represent SEM.

Gelatinase, a type of metalloproteinase, activity was determined for representative elapid and viper snake venoms, *N. kaouthia* and *D. russelii*, respectively, as well as *A. alata* (Figure 4). Each venom demonstrated activity over an 8 h incubation period (Figure 4A–C). While *A. alata* venom reached a gelatinase activity maximum at 2 h (Figure 4C), both snake venoms’ activity continued past 8 h (Figure 4A,B).

### 2.4. Inhibition of Gelatinase Activities

**Figure 5:**
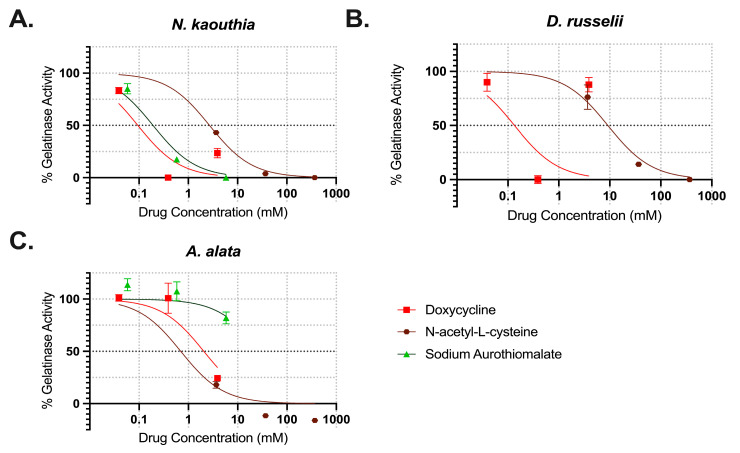
Summary of gelatinase activity inhibition by small-molecule drugs. The % gelatinase activity was determined for selected inhibitors at 60 min in the presence of (**A**) *N. kaouthia* (5 mg/mL), (**B**) *D. russelii* (1 mg/mL), and (**C**) *A. alata* (0.67 mg/mL). Each point represents the mean of three replicates and the error bars represent SEM.

Three of the literature-reported MMP inhibitors were evaluated: doxycycline, sodium aurothiomalate, and *N*-acetyl-L-cysteine. The gelatinase activity of *N. kaouthia*, *D. russelii*, and *A. alata* at the 60 min time point was normalized and then plotted as a function of inhibitor concentrations (Figure 5). A non-linear regression curve was used to estimate the best-fit value for the concentrations required to reduce gelatinase activity by 50% for each inhibitor spanning the 0–100% activity range. Doxycycline reduced gelatinase activity by ~50% at 2.1 mM (95% CI 1.3–3.0%) for *A. alata* (Figure 5C) and completely inhibited both snake venoms below 1 mM (Figure 5A,B). Sodium aurothiomalate reduced *N. kaouthia* gelatinase activity by 50% at 0.2 mM (95% CI 0.16 to 0.23 mM), but did not effectively reduce *A. alata* or *D. russelii* venoms over the selected concentration range. At 9.2 (95% CI 7.2 to 11.7 mM), *N*-acetyl-L-cysteine reduced *D. russelii* activity by 50% and completely inhibited both *N. kaouthia* and *A. alata* gelatinase activity.

## 3. Discussion

Across diverse animal phyla, some venomous species share highly conserved enzyme classes and proteins in their venom arsenal [82]. Specifically, phospholipases, metalloproteinases, and pore-forming proteins are common constituents, likely due to their importance in prey capture and digestion, as well as defense [40,43,44,45,48,58]. Envenomations represent a significant public health threat due to the potent pathophysiological effects of these venom arsenals; such injuries predominantly occur in rural or austere environments in the tropics [1,2,3,4,5,6,7,8]. For example, snakebite envenoming results in 80,000–138,000 deaths annually and causes three times as many people to suffer permanent disabilities or other morbidities due to the tissue-destroying effects of venom components [83]. Traditionally, snakebite envenomation has been treated with species-specific antivenoms, effectively inhibiting selected neurotoxins with life-saving outcomes [83,84]. However, issues with accessibility and affordability limit this approach for certain high-risk populations [63,64,65]. Further, venom enzyme-driven tissue damage and morbidity can still occur even with prompt and appropriate administration of antivenoms [57,66]. Investigation into small-molecular inhibitors for conserved venom components provides a low-cost, accessible alternative for envenomation treatment to improve victim outcomes. Further, because the targeted venom components are conserved in diverse phyla, there is potential for broader use. Here, we evaluated the ability of small-molecule inhibitors of snake venom PLA_2_ and MMP against *A. alata* venom activities.

Doxycycline has previously been shown to inhibit MMP and PLA_2_ activity in snake venoms [75]. The predicted mechanism of inhibition differs between the enzymes; doxycycline interferes with substrate binding at the active site of phospholipases [85,86] and has zinc chelation properties that interrupt metalloproteinase activity [87]. Our results are consistent with the literature, demonstrating the ability of doxycycline to inhibit both *N. kaouthia* and *D. russelii* PLA_2_ and gelatinase activities in the 0.1–1 mM range. Further, we demonstrate doxycycline can inhibit *A. alata* PLA_2_ and MMP activity (approximately 50% reduction at 0.1 mM (95% CI 0.06–0.15) and 2.1 mM (95% CI 1.4–3.0), respectively). Bee venom PLA_2_ was completely inhibited by 0.1 mM across all time points evaluated (Figure 2I). Our findings support the efficacy of doxycycline in inhibiting PLA_2_ and MMP activities for numerous venomous species.

Varespladib is currently in a phase II clinical trial for snakebite treatment [67]. Here, we evaluated the ability of varespladib to inhibit *A. alata* venom PLA_2_ activity in addition to bee venom PLA_2_ and representative elapid and viper venoms. As expected, varespladib completely inhibited both snake venoms over the concentration range selected (0.0013–0.25 mM) (Figure 2B,C and Figure 3B,C). Inhibition of at least 50% was not achieved in either *A. alata* (Figure 2D and Figure 3D) or bee venom PLA_2_ (Figure 2A and Figure 3A). Higher concentration ranges could be tested. Alternatively, the active sites of the *A. alata* or bee venom PLA_2_ may not be fully affected by varespladib. However, in vivo models have demonstrated varespladib’s ability to protect against systemic toxicity from hymenopteran venoms, despite its low potency in vitro [88,89]. This effect may be due to the inhibition of host sPLA_2_, rather than the PLA_2_ from the venom. Post-sting inflammation is amplified by activation of host sPLA_2_ [89]. Varespladib is predicted to block snake venom PLA_2_ interaction with allosteric activator molecules via binding in the hydrophobic channel [90]. Thus, there are important structural differences between sPLA_2_ subgroups that contribute to their responsiveness to small-molecule inhibitors. Bee venom PLA_2_ belongs to group III of sPLA_2_, whereas viper and elapid PLA_2_ fall in groups IA and IIA/IIB, respectively [91,92]. The molecular structure of *A. alata* PLA_2_, as well as cnidaria, is not yet elucidated. Amino acid sequencing and structural determination of venom PLA_2_ pose a critical future direction to develop optimal inhibitors or modify extant small molecules for improved efficacy.

Divalent copper, in the form of copper gluconate, has been identified as a potent inhibitor of pore-forming toxins from jellyfish (~30 µM) and fire-ants (~3 µM) [62]. It has been postulated that divalent copper interrupts the calcium-dependent self-assembly of cnidarian porins. Investigation of copper gluconate in this study demonstrated broad-acting inhibition of PLA_2_ in *A. alata*, bee, and snake venoms, although at higher concentrations than to inhibit pore-forming activity (mM vs. µM). The concentration required to reduce activity by 50% ranged from 9.0 mM (95% CI 8.0 to 10.2 mM) for bee venom to 14 mM (95% CI 11.5 to 16.8 mM) for *A. alata*.

*N*-acetyl-L-cysteine and sodium aurothiomalate have reportedly reduced adverse outcomes in rat and mouse models of snake envenomation [72,73,74]. Here, we evaluated *N*-acetyl-L-cysteine’s ability to inhibit both PLA_2_ and gelatinase activity. While effective at reducing each of the venoms’ PLA_2_ activity, a higher concentration (30–60 mM) was required to observe 50% inhibition as compared to the other small molecules (Figure 3A–E). However, 4 mM *N*-acetyl-L-cysteine reduced both *N. kaouthia* and *A. alata* gelatinase activity by over 50%. Sodium aurothiomalate did not inhibit PLA_2_ activity in any of the venoms evaluated. While it reduced gelatinase activity in *N. kaouthia* venom, it was not effective for either *A. alata* or *D. russelii*.

The venom of *A. alata* possesses robust pore-forming activity, requiring 10 ng/mL concentrations to reach the 50% hemolytic unit (HU_50_) value (amount of protein required to lyse 50% of RBC in 1 mL of a 1% RBC solution at 37 °C in 1 h) [58]. Further studies have shown that the time-course (within minutes) for an acute lethal dose of the total venom was fully recapitulated by the isolated porin alone [93]. For these reasons, identifying inhibitors of the pore-forming activity has been the main focus for therapeutic development of envenomation and first aid. In this study, we demonstrate phospholipase and gelatinase activity from complete *A. alata* venom [58] for the first time. The specific activities of the cubozoan venom phospholipase (Figure 1D) and gelatinase (Figure 4C) were far lower than the hemolytic activity (µg/mL rather than ng/mL). While less potent, these components likely contribute to post-sting inflammation and tissue damage. Thus, agents capable of inhibiting these activities could be useful in improving sting outcomes. Numerous studies have evaluated metalloproteinase activity in scyphozoans, as well as potential inhibitors [94]. Batimastat has been shown to mitigate dermatological and oedematogenic symptoms of scyphozoan envenomation [77,95]. Whether batimastat (or related marimastat) and copper gluconate can also inhibit cubozoan MMP remains to be determined.

We have shown that while some small molecules can inhibit across multiple animal phyla, or different enzyme classes, they often require higher concentrations to exert effects. There is the potential for cytotoxic effects at these levels. For instance, while copper gluconate can potently inhibit pore-forming proteins at the 3–30 µM range, we found that 5–14 mM concentrations were required to reduce PLA_2_ activity. Continuous exposure to ~1 mM copper gluconate significantly reduced Vero E6 cell viability over a 24–72 h period [96]. Similarly, snake venom PLA_2_ is completely inhibited by nM concentrations of varespladib [59], yet bee and jellyfish venom require more than 0.3 mM for inhibition. The maximum tolerated concentration of varespladib for human epidermal keratinocytes (HaCaT) was determined to be 256 µM [61], far beyond the concentration requirement for bee and jellyfish venoms. Doxycycline, in contrast, did not exert any toxic effects on HaCaT cells at 675 mM [97], which exceeds the efficacious concentrations we have reported. These in vitro toxicity studies continuously subject cells to the described molecule for 24 to 72 h. Ideally, in the case of envenomation, a small molecule or combination would be applied directly to the sting or bite site at a high dose for a short period. This could minimize the potential toxicity due to prolonged exposures.

Small-molecular inhibitors could provide alternatives, or augment, the current standard of care for envenomations. Ideal characteristics include efficacy, safety, thermostability, and cost-effectiveness. Future directions of this study include combinatorial analysis of the described small molecules, including doxycycline, varespladib, marimastat, copper gluconate, and *n*-acetyl-l-cysteine. Next steps also include examining the translational efficacy from solution-based assays to tissue models and in vivo assays to determine the applicability of potential therapeutics. Specifically planned studies include the use of an in vivo piglet model [98,99] with live tentacle stings to assess the inhibition of downstream pathophysiological effects, where these can be continuously monitored and the contribution of host-derived components to the complex sequelae can also be evaluated.

## 4. Materials and Methods

### 4.1. Venom Preparation

*Alatina alata* venom preparation followed the Yanagihara and Shohet (2012) methodology [58]. Briefly, whole animals were collected in Waikiki, Hawaii, USA, and their tentacles were excised into 1 M trisodium citrate at 1:4 (*v*:*v*). The tentacle–citrate solution was kept at 4 °C with gentle rotation to allow intact cnidae to shed off their tentacles into the solution. The rotation continued until 95% recovery of intact cnidae was accomplished, quantified microscopically with a hemocytometer. Then, the solution was sieved and centrifuged (400× *g*, 20 min, 4 °C). Pellets were resuspended in 4 °C 1 M trisodium citrate and washed again (250× *g*, 20 min, 4 °C). The pellets were resuspended in ice-cold deionized (18.2 MΩ) water at in 1:0.5 (*v*:*v*) and immediately loaded into a chilled French Press 20 K pressure cell (SLM Aminco FA-078 FRENCH Pressure Cell Press 115 V). The pressure cell was set to 12,000 psi, with a flow rate of 30 drops/min of lysate. The solution was recycled through the instrument until >90% cnidae rupture was detected via microscopy. The final lysate was centrifuged (12,000× *g*, 5 min, 4 °C) to pellet the collagen capsule and structural components of the cnidae. The supernatant, cnidae venom content, was then aliquoted and snap-frozen in liquid nitrogen. Samples were kept at −80 °C until use. Snake venom from *Daboia russelii* and *Naja kaouthia* was purchased from Sigma Aldrich (V2501 and V9125, Saint Louis, MO, USA). The snake venoms were reconstituted in 1× PLA_2_ reaction buffer (Invitrogen™, E10217, Eugene, OR, USA) or 1× gelatinase reaction buffer (Invitrogen™, E12055, Eugene, OR, USA) and kept at −80 °C until use. *Apis mellifera* venom PLA_2_ was included in the Invitrogen EnzChek^®^ Phospholipase A_2_ Assay Kit.

### 4.2. In Vitro Experiments

The protein concentration of *Alatina alata* venom sister aliquots was determined by Bradford assay. Briefly, Pierce™ Bovine Serum Albumin (BSA) standard ampules, at 2 mg/mL (ThermoScientific, Cat#23209, Rockford, IL, USA), were serially diluted in deionized (18.2 MΩ) water, and then combined with Protein Assay Reagent Dye (Bio-Rad, Cat#5000006, Hercules, CA, USA) and incubated at room temperature for 5 min. A standard curve was produced by measuring the absorbance at 595 nm. *A. alata* venom was similarly serially diluted in deionized water, and then combined with the Protein Assay Reagent Dye and incubated at room temperature for 5 min. The absorbance was measured at 595 nm and protein concentration calculated using the standard curve.

Small-molecule inhibitors were purchased and stored per manufacturers’ recommendations. Doxycycline hyclate (Sigma-Aldrich, D9891, Saint Louis, MO, USA), *N*-acetyl-L-cysteine (Sigma-Aldrich, A7250, Saint Louis, MO, USA), sodium aurothiomalate (Sigma-Aldrich, 157201, Saint Louis, MO, USA), copper gluconate (Spectrum Chemical, C1329, New Brunswick, NJ, USA), varespladib (Sigma-Aldrich, SML1100, Saint Louis, MO, USA), and marimastat (Sigma-Aldrich, M2699, Saint Louis, MO, USA) were purchased from listed vendors.

Phospholipase A_2_ activity and inhibition were determined following the EnzChek™ Phospholipase A_2_ Assay Kit (Invitrogen™, E10217, Eugene, OR, USA) specifications. Briefly, 50 µL of each venom prepared in 1× reaction buffer (50 mM Tris-HCl, 100 mM NaCl, 1 mM CaCl_2_, pH 8.9) at 2× the desired final concentration was combined with 50 µL of lipid mix (Red/Gren BODPIY^®^ PC-A2, dioleoylphosphatidylcholine, dioleoylphosphatidylglycerol, DMSO) in quintuplicate in a 96-well plate. For the inhibition assays, 50 µL of venom was mixed into the lipid substrate, and then immediately aliquoted into a 96-well plate containing 50 µL of small molecules prepared in 1× reaction buffer at 2× the final concentration. Varespladib was prepared in DMSO prior to dilution in 1× reaction buffer. The venom concentration was determined by the preliminary activity assay, at values where there was high PLA_2_ activity relative to the negative control but without complete substrate depletion at 60 min (approximately 50% of maximal MFI). The plate was incubated protected from the light at room temperature (~20 °C), with the mean fluorescence intensity (MFI) measured (485 excitation, 535 emission) at set time intervals. The initial reading was performed immediately after combining the venom and substrate, with or without inhibitors. Additional fluorescence readings were performed at 15 min increments up to one hour. Reaction buffer alone served as the negative control and honeybee venom PLA_2_ (5 U/mL) served as the positive control. Additional negative controls included each small-molecule inhibitor in its respective buffer, without the addition of venom.

Gelatinase activity and inhibition were determined following the EnzChek™ Gelatinase/Collagenase Assay Kit (Invitrogen™, E12055, Eugene, OR, USA) specifications. For the gelatinase activity assay, 100 µL of each venom at 2× the desired final concentration was added to 80 µL 1× reaction buffer (0.05 M Tris-HCl, 0.15 M NaCl, 5 mM CaCl_2_, 0.2 mM sodium azide, pH 7.6) and 20 µL of DQ gelatin (from pig skin, with fluorescein conjugate) in sextuplicate in a 96-well plate. For the inhibition assays, the venom concentration was selected to provide high gelatinase activity in comparison to the negative control, but without depleting the substrate (~50% maximal MFI). Small-molecule inhibitors were prepared at 2.5× the desired final concentration in 1× reaction buffer. Then, 100 µL of venom at 2× the desired final concentration was combined with 80 µL of the selected small molecule, followed by immediate addition of 20 µL of DQ gelatin mix in triplicate in a 96-well plate. The plates were incubated at room temperature (~20 °C), protected from the light, for eight hours, while taking mean fluorescence intensity (MFI) readings (485 excitation, 535 emission) at set intervals. Reaction buffer alone served as the negative control and *Clostridium histolyticum* (0.2 U/mL) served as the positive control. Additional negative controls included each small-molecule inhibitor in its respective buffer, without the addition of venom.

### 4.3. Statistical Analysis

Raw mean fluorescence intensity (MFI) readings were imported to GraphPad Prism (Version 10.4.0) for graphing, analysis, and concentrations required to reduce activity by 50% calculations. Results were expressed as mean ± standard error of the mean (SEM). An ordinary two-way ANOVA and Dunnett’s multiple comparisons test were performed for the PLA_2_ inhibition experiment. At each time point, the inhibitors were compared to venom alone and the significance, in terms of *p*-value, was calculated (*p*-values: * < 0.05, ** < 0.01, *** < 0.001, **** ≤ 0.0001). For the determination of the concentration required to reduce venom activity by 50%, the MFI values were first normalized such that venom alone represented 100% activity and reaction buffer represented 0% activity. The concentration for 50% reduction in enzymatic activity values were calculated as best-fit values fitting a non-linear regression curve (least squares regression method, with the maximum number of iterations set to 1000, and no weighting), using the [inhibitor] vs. normalized response model on GraphPad Prism, with a 95% confidence interval.

## Data Availability

The original contributions presented in this study are included in the article/Supplementary Material. Further inquiries can be directed to the corresponding author(s).

## Supplementary Materials

The following supporting information can be downloaded at https://www.mdpi.com/article/10.3390/toxins17040188/s1: Table S1. Dunnett’s multiple comparisons test for PLA_2_ inhibition Figure S1. Marimastat and sodium aurothiomalate inhibition of PLA_2_ activity.

## Abbreviations

The following abbreviations are used in this manuscript:

MDPI	Multidisciplinary Digital Publishing Institute
MMP	Matrix metalloproteinase
PLA_2_	Phospholipase A2
SSS	Snakebite Severity Score
