# Supplementary material for: Assessing the Utility of Broad-Acting Inhibitors as Therapeutics in Diverse Venoms
Source: Toxins (Basel). 2025 Apr 8;17(4):188. doi: 10.3390/toxins17040188 (PMC12031005; doi:10.3390/toxins17040188)
Supplement: Supplementary file 1 [file toxins-17-00188-s001.zip › toxins-3532241-supplementary.pdf]

Supplementary Materials: Assessing the Utility of Broad-Acting Inhibitors as Therapeutics in Diverse Venoms

Raechel Kadler, Breanna Morrison and Angel Anne Yanagihara

Table S1. Dunnett's Multiple Comparisons Test for PLA_2 Inhibition

Dunnett's multiple comparisons test	Mean Diff.	95.00% CI of diff.	Below threshold?	Summary	Adjusted P Value
**Figure 2A - Bee Venom PLA2**					
**Time point = 0 mins**					
Venom Alone vs. 0.0013 mM VA	479542	157367 to 801717	Yes	**	0.0014
Venom Alone vs. 0.0053 mM VA	193766	-128409 to 515941	No	ns	0.3955
Venom Alone vs. 0.026 mM VA	-18635	-340810 to 303540	No	ns	>0.9999
Venom Alone vs. 0.13 mM VA	38759	-283416 to 360934	No	ns	0.998
Venom Alone vs. 0.26 mM VA	16735	-305440 to 338910	No	ns	>0.9999
**Time point = 15 mins**					
Venom Alone vs. 0.0013 mM VA	458537	136362 to 780712	Yes	**	0.0023
Venom Alone vs. 0.0053 mM VA	146922	-175253 to 469097	No	ns	0.6511
Venom Alone vs. 0.026 mM VA	-77651	-399826 to 244524	No	ns	0.9568
Venom Alone vs. 0.13 mM VA	25198	-296977 to 347373	No	ns	0.9997
Venom Alone vs. 0.26 mM VA	-97985	-420160 to 224190	No	ns	0.8962
**Time point = 30 mins**					
Venom Alone vs. 0.0013 mM VA	293186	-28989 to 615361	No	ns	0.0862
Venom Alone vs. 0.0053 mM VA	118307	-203868 to 440482	No	ns	0.8071
Venom Alone vs. 0.026 mM VA	-108340	-430515 to 213835	No	ns	0.8539
Venom Alone vs. 0.13 mM VA	251	-321924 to 322426	No	ns	>0.9999
Venom Alone vs. 0.26 mM VA	-136014	-458189 to 186161	No	ns	0.713
**Time point = 45 mins**					
Venom Alone vs. 0.0013 mM VA	228164	-94011 to 550339	No	ns	0.2491
Venom Alone vs. 0.0053 mM VA	111555	-210620 to 433730	No	ns	0.8394
Venom Alone vs. 0.026 mM VA	-101638	-423813 to 220537	No	ns	0.8821
Venom Alone vs. 0.13 mM VA	119	-322056 to 322294	No	ns	>0.9999
Venom Alone vs. 0.26 mM VA	-123639	-445814 to 198536	No	ns	0.7799
**Time point = 60 mins**					
Venom Alone vs. 0.0013 mM VA	215865	-106310 to 538040	No	ns	0.2963
Venom Alone vs. 0.0053 mM VA	101515	-220660 to 423690	No	ns	0.8826
Venom Alone vs. 0.026 mM VA	-92939	-415114 to 229236	No	ns	0.9142
Venom Alone vs. 0.13 mM VA	1079	-321096 to 323254	No	ns	>0.9999
Venom Alone vs. 0.26 mM VA	-125862	-448037 to 196313	No	ns	0.7683
**Figure 2B - *Naja kaouthia***					
**Time point = 0 mins**					
Venom Alone vs. 0.0013 mM VA	169052	145526 to 192578	Yes	****	<0.0001
Venom Alone vs. 0.0053 mM VA	172165	148639 to 195691	Yes	****	<0.0001
Venom Alone vs. 0.026 mM VA	157713	134187 to 181239	Yes	****	<0.0001
Venom Alone vs. 0.13 mM VA	148342	124816 to 171868	Yes	****	<0.0001
**Dunnett's multiple comparisons test**	**Mean Diff.**	**95.00% CI of diff.**	**Below threshold?**	**Summary**	**Adjusted P Value**

Venom Alone vs. 0.26 mM VA	143396	119870 to 166922	Yes	****	<0.0001
**Time point = 15 mins**					
Venom Alone vs. 0.0013 mM VA	570287	546761 to 593813	Yes	****	<0.0001
Venom Alone vs. 0.0053 mM VA	581412	557886 to 604938	Yes	****	<0.0001
Venom Alone vs. 0.026 mM VA	570972	547446 to 594498	Yes	****	<0.0001
Venom Alone vs. 0.13 mM VA	555550	532024 to 579076	Yes	****	<0.0001
Venom Alone vs. 0.26 mM VA	552005	528479 to 575531	Yes	****	<0.0001
**Time point = 30 mins**					
Venom Alone vs. 0.0013 mM VA	731216	707690 to 754742	Yes	****	<0.0001
Venom Alone vs. 0.0053 mM VA	744893	721367 to 768419	Yes	****	<0.0001
Venom Alone vs. 0.026 mM VA	735668	712142 to 759194	Yes	****	<0.0001
Venom Alone vs. 0.13 mM VA	720112	696586 to 743638	Yes	****	<0.0001
Venom Alone vs. 0.26 mM VA	717940	694414 to 741466	Yes	****	<0.0001
**Time point = 45 mins**					
Venom Alone vs. 0.0013 mM VA	821780	798254 to 845306	Yes	****	<0.0001
Venom Alone vs. 0.0053 mM VA	839549	816023 to 863075	Yes	****	<0.0001
Venom Alone vs. 0.026 mM VA	830971	807445 to 854497	Yes	****	<0.0001
Venom Alone vs. 0.13 mM VA	814717	791191 to 838243	Yes	****	<0.0001
Venom Alone vs. 0.26 mM VA	811798	788272 to 835324	Yes	****	<0.0001
**Time point = 60 mins**					
Venom Alone vs. 0.0013 mM VA	838918	815392 to 862444	Yes	****	<0.0001
Venom Alone vs. 0.0053 mM VA	860218	836692 to 883744	Yes	****	<0.0001
Venom Alone vs. 0.026 mM VA	852807	829281 to 876333	Yes	****	<0.0001
Venom Alone vs. 0.13 mM VA	836060	812534 to 859586	Yes	****	<0.0001
Venom Alone vs. 0.26 mM VA	834581	811055 to 858107	Yes	****	<0.0001
**Figure 2C - *Daboia russelii***					
**Time point = 0 mins**					
Venom Alone vs. 0.0013 mM VA	464709	422084 to 507334	Yes	****	<0.0001
Venom Alone vs. 0.0053 mM VA	490372	447747 to 532997	Yes	****	<0.0001
Venom Alone vs. 0.026 mM VA	490650	448025 to 533275	Yes	****	<0.0001
Venom Alone vs. 0.13 mM VA	512232	469607 to 554857	Yes	****	<0.0001
Venom Alone vs. 0.26 mM VA	497091	454466 to 539716	Yes	****	<0.0001
**Time point = 15 mins**					
Venom Alone vs. 0.0013 mM VA	799902	757277 to 842527	Yes	****	<0.0001
Venom Alone vs. 0.0053 mM VA	825868	783243 to 868493	Yes	****	<0.0001
Venom Alone vs. 0.026 mM VA	832505	789880 to 875130	Yes	****	<0.0001
Venom Alone vs. 0.13 mM VA	843434	800809 to 886059	Yes	****	<0.0001
**Dunnett's multiple comparisons test**	**Mean Diff.**	**95.00% CI of diff.**	**Below threshold?**	**Summary**	**Adjusted P Value**
Venom Alone vs. 0.26 mM VA	842254	799629 to 884879	Yes	****	<0.0001
**Time point = 30 mins**					
Venom Alone vs. 0.0013 mM VA	851879	809254 to 894504	Yes	****	<0.0001
Venom Alone vs. 0.0053 mM VA	880627	838002 to 923252	Yes	****	<0.0001

Venom Alone vs. 0.026 mM VA	884849	842224 to 927474	Yes	****	<0.0001
Venom Alone vs. 0.13 mM VA	899291	856666 to 941916	Yes	****	<0.0001
Venom Alone vs. 0.26 mM VA	898792	856167 to 941417	Yes	****	<0.0001
**Time point = 45 mins**					
Venom Alone vs. 0.0013 mM VA	850777	808152 to 893402	Yes	****	<0.0001
Venom Alone vs. 0.0053 mM VA	882214	839589 to 924839	Yes	****	<0.0001
Venom Alone vs. 0.026 mM VA	887085	844460 to 929710	Yes	****	<0.0001
Venom Alone vs. 0.13 mM VA	902102	859477 to 944727	Yes	****	<0.0001
Venom Alone vs. 0.26 mM VA	901366	858741 to 943991	Yes	****	<0.0001
**Time point = 60 mins**					
Venom Alone vs. 0.0013 mM VA	846013	803388 to 888638	Yes	****	<0.0001
Venom Alone vs. 0.0053 mM VA	880302	837677 to 922927	Yes	****	<0.0001
Venom Alone vs. 0.026 mM VA	884922	842297 to 927547	Yes	****	<0.0001
Venom Alone vs. 0.13 mM VA	900430	857805 to 943055	Yes	****	<0.0001
Venom Alone vs. 0.26 mM VA	899346	856721 to 941971	Yes	****	<0.0001
**Figure 2D - *Alatina alata***					
**Time point = 0 mins**					
Venom Alone vs. 0.0013 mM VA	17609	-14659 to 49877	No	ns	0.4881
Venom Alone vs. 0.0053 mM VA	22911	-9357 to 55179	No	ns	0.2469
Venom Alone vs. 0.026 mM VA	19313	-12955 to 51581	No	ns	0.4
Venom Alone vs. 0.13 mM VA	21736	-10532 to 54004	No	ns	0.2916
Venom Alone vs. 0.26 mM VA	20951	-11317 to 53219	No	ns	0.3244
**Time point = 15 mins**					
Venom Alone vs. 0.0013 mM VA	33527	1260 to 65795	Yes	*	0.039
Venom Alone vs. 0.0053 mM VA	48997	16730 to 81265	Yes	**	0.0011
Venom Alone vs. 0.026 mM VA	54942	22674 to 87210	Yes	***	0.0002
Venom Alone vs. 0.13 mM VA	63769	31502 to 96037	Yes	****	<0.0001
Venom Alone vs. 0.26 mM VA	69807	37540 to 102075	Yes	****	<0.0001
**Time point = 30 mins**					
Venom Alone vs. 0.0013 mM VA	62890	30622 to 95157	Yes	****	<0.0001
Venom Alone vs. 0.0053 mM VA	88233	55965 to 120501	Yes	****	<0.0001
Venom Alone vs. 0.026 mM VA	93962	61694 to 126230	Yes	****	<0.0001
Venom Alone vs. 0.13 mM VA	107891	75623 to 140158	Yes	****	<0.0001
**Dunnett's multiple comparisons test**	**Mean Diff.**	**95.00% CI of diff.**	**Below threshold?**	**Summary**	**Adjusted P Value**
Venom Alone vs. 0.26 mM VA	117233	84966 to 149501	Yes	****	<0.0001
**Time point = 45 mins**					
Venom Alone vs. 0.0013 mM VA	74028	41760 to 106295	Yes	****	<0.0001
Venom Alone vs. 0.0053 mM VA	106296	74029 to 138564	Yes	****	<0.0001
Venom Alone vs. 0.026 mM VA	110459	78192 to 142727	Yes	****	<0.0001
Venom Alone vs. 0.13 mM VA	126563	94295 to 158831	Yes	****	<0.0001
Venom Alone vs. 0.26 mM VA	138428	106160 to 170695	Yes	****	<0.0001
**Time point = 60 mins**					

Venom Alone vs. 0.0013 mM VA	76095	43828 to 108363	Yes	****	<0.0001
Venom Alone vs. 0.0053 mM VA	114860	82592 to 147127	Yes	****	<0.0001
Venom Alone vs. 0.026 mM VA	117464	85196 to 149732	Yes	****	<0.0001
Venom Alone vs. 0.13 mM VA	138281	106014 to 170549	Yes	****	<0.0001
Venom Alone vs. 0.26 mM VA	155360	123092 to 187628	Yes	****	<0.0001
**Figure 2E - Bee Venom PLA2**					
**Time point = 0 mins**					
Venom Alone vs. 0.2 mM Dox	1843068	1678407 to 2007729	Yes	****	<0.0001
Venom Alone vs. 0.1 mM Dox	1550962	1386301 to 1715623	Yes	****	<0.0001
Venom Alone vs. 0.4 mM Dox	2174174	2009513 to 2338835	Yes	****	<0.0001
Venom Alone vs. 2 mM Dox	2526280	2361620 to 2690941	Yes	****	<0.0001
Venom Alone vs. 20 mM Dox	2223819	2059158 to 2388480	Yes	****	<0.0001
**Time point = 15 mins**					
Venom Alone vs. 0.2 mM Dox	4787936	4623275 to 4952597	Yes	****	<0.0001
Venom Alone vs. 0.1 mM Dox	4450599	4285938 to 4615260	Yes	****	<0.0001
Venom Alone vs. 0.4 mM Dox	5120386	4955725 to 5285047	Yes	****	<0.0001
Venom Alone vs. 2 mM Dox	5319798	5155137 to 5484458	Yes	****	<0.0001
Venom Alone vs. 20 mM Dox	4991682	4827021 to 5156343	Yes	****	<0.0001
**Time point = 30 mins**					
Venom Alone vs. 0.2 mM Dox	5357043	5192382 to 5521704	Yes	****	<0.0001
Venom Alone vs. 0.1 mM Dox	4940768	4776107 to 5105429	Yes	****	<0.0001
Venom Alone vs. 0.4 mM Dox	5644701	5480041 to 5809362	Yes	****	<0.0001
Venom Alone vs. 2 mM Dox	5793138	5628478 to 5957799	Yes	****	<0.0001
Venom Alone vs. 20 mM Dox	5461399	5296738 to 5626060	Yes	****	<0.0001
**Time point = 45 mins**					
**Dunnett's multiple comparisons test**	**Mean Diff.**	**95.00% CI of diff.**	**Below threshold?**	**Summary**	**Adjusted P Value**
Venom Alone vs. 0.2 mM Dox	5438201	5273540 to 5602862	Yes	****	<0.0001
Venom Alone vs. 0.1 mM Dox	4987617	4822956 to 5152278	Yes	****	<0.0001
Venom Alone vs. 0.4 mM Dox	5687604	5522944 to 5852265	Yes	****	<0.0001
Venom Alone vs. 2 mM Dox	5795844	5631184 to 5960505	Yes	****	<0.0001
Venom Alone vs. 20 mM Dox	5481579	5316918 to 5646240	Yes	****	<0.0001
**Time point = 60 mins**					
Venom Alone vs. 0.2 mM Dox	5439386	5274725 to 5604047	Yes	****	<0.0001
Venom Alone vs. 0.1 mM Dox	4955968	4791307 to 5120629	Yes	****	<0.0001
Venom Alone vs. 0.4 mM Dox	5659212	5494551 to 5823872	Yes	****	<0.0001
Venom Alone vs. 2 mM Dox	5736003	5571343 to 5900664	Yes	****	<0.0001
Venom Alone vs. 20 mM Dox	5442680	5278019 to 5607341	Yes	****	<0.0001
**Figure 2F - *Naja kaouthia***					
**Time point = 0 mins**					
Venom Alone vs. 0.0013 mM VA	190332	98415 to 282249	Yes	****	<0.0001
Venom Alone vs. 0.0053 mM VA	187980	85213 to 290747	Yes	****	<0.0001
Venom Alone vs. 0.026 mM VA	225432	133515 to 317349	Yes	****	<0.0001

Venom Alone vs. 0.13 mM VA	299158	207241 to 391075	Yes	****	<0.0001
Venom Alone vs. 0.26 mM VA	244095	152178 to 336012	Yes	****	<0.0001
**Time point = 15 mins**					
Venom Alone vs. 0.0013 mM VA	529853	437936 to 621770	Yes	****	<0.0001
Venom Alone vs. 0.0053 mM VA	568414	465647 to 671180	Yes	****	<0.0001
Venom Alone vs. 0.026 mM VA	663627	571710 to 755544	Yes	****	<0.0001
Venom Alone vs. 0.13 mM VA	777419	685501 to 869336	Yes	****	<0.0001
Venom Alone vs. 0.26 mM VA	700204	608286 to 792121	Yes	****	<0.0001
**Time point = 30 mins**					
Venom Alone vs. 0.0013 mM VA	627745	535827 to 719662	Yes	****	<0.0001
Venom Alone vs. 0.0053 mM VA	684267	581501 to 787034	Yes	****	<0.0001
Venom Alone vs. 0.026 mM VA	816335	724418 to 908252	Yes	****	<0.0001
Venom Alone vs. 0.13 mM VA	949680	857763 to 1041597	Yes	****	<0.0001
Venom Alone vs. 0.26 mM VA	866569	774651 to 958486	Yes	****	<0.0001
**Time point = 45 mins**					
Venom Alone vs. 0.0013 mM VA	655511	563594 to 747429	Yes	****	<0.0001
Venom Alone vs. 0.0053 mM VA	732638	629871 to 835404	Yes	****	<0.0001
Venom Alone vs. 0.026 mM VA	893125	801208 to 985043	Yes	****	<0.0001
**Dunnett's multiple comparisons test**	**Mean Diff.**	**95.00% CI of diff.**	**Below threshold?**	**Summary**	**Adjusted P Value**
Venom Alone vs. 0.13 mM VA	1047170	955252 to 1139087	Yes	****	<0.0001
Venom Alone vs. 0.26 mM VA	962527	870610 to 1054445	Yes	****	<0.0001
**Time point = 60 mins**					
Venom Alone vs. 0.0013 mM VA	633929	542012 to 725847	Yes	****	<0.0001
Venom Alone vs. 0.0053 mM VA	721377	618611 to 824144	Yes	****	<0.0001
Venom Alone vs. 0.026 mM VA	899657	807739 to 991574	Yes	****	<0.0001
Venom Alone vs. 0.13 mM VA	1067046	975128 to 1158963	Yes	****	<0.0001
Venom Alone vs. 0.26 mM VA	980399	888481 to 1072316	Yes	****	<0.0001
**Figure 2G - *Daboia russelii***					
**Time point = 0 mins**					
Venom Alone vs. 0.1 mM Dox	272894	234633 to 311155	Yes	****	<0.0001
Venom Alone vs. 0.2 mM Dox	386602	348341 to 424863	Yes	****	<0.0001
Venom Alone vs. 0.4 mM Dox	503188	464927 to 541449	Yes	****	<0.0001
Venom Alone vs. 2 mM Dox	660989	622728 to 699250	Yes	****	<0.0001
Venom Alone vs. 20 mM Dox	615571	577310 to 653832	Yes	****	<0.0001
**Time point = 15 mins**					
Venom Alone vs. 0.1 mM Dox	373488	335227 to 411749	Yes	****	<0.0001
Venom Alone vs. 0.2 mM Dox	561773	523512 to 600034	Yes	****	<0.0001
Venom Alone vs. 0.4 mM Dox	839767	801506 to 878028	Yes	****	<0.0001
Venom Alone vs. 2 mM Dox	1023106	984845 to 1061367	Yes	****	<0.0001
Venom Alone vs. 20 mM Dox	974125	935864 to 1012386	Yes	****	<0.0001
**Time point = 30 mins**					
Venom Alone vs. 0.1 mM Dox	304334	266073 to 342595	Yes	****	<0.0001

Venom Alone vs. 0.2 mM Dox	523660	485399 to 561921	Yes	****	<0.0001
Venom Alone vs. 0.4 mM Dox	868006	829745 to 906267	Yes	****	<0.0001
Venom Alone vs. 2 mM Dox	1078070	1039809 to 1116331	Yes	****	<0.0001
Venom Alone vs. 20 mM Dox	1041410	1003149 to 1079671	Yes	****	<0.0001
**Time point = 45 mins**					
Venom Alone vs. 0.1 mM Dox	252825	214564 to 291086	Yes	****	<0.0001
Venom Alone vs. 0.2 mM Dox	471540	433279 to 509801	Yes	****	<0.0001
Venom Alone vs. 0.4 mM Dox	855514	817253 to 893775	Yes	****	<0.0001
Venom Alone vs. 2 mM Dox	1082825	1044564 to 1121086	Yes	****	<0.0001
Venom Alone vs. 20 mM Dox	1049447	1011186 to 1087708	Yes	****	<0.0001
**Time point = 60 mins**					
Venom Alone vs. 0.1 mM Dox	225761	187500 to 264022	Yes	****	<0.0001
**Dunnett's multiple comparisons test**	**Mean Diff.**	**95.00% CI of diff.**	**Below threshold?**	**Summary**	**Adjusted P Value**
Venom Alone vs. 0.2 mM Dox	433683	395422 to 471944	Yes	****	<0.0001
Venom Alone vs. 0.4 mM Dox	843341	805080 to 881602	Yes	****	<0.0001
Venom Alone vs. 2 mM Dox	1089352	1051091 to 1127613	Yes	****	<0.0001
Venom Alone vs. 20 mM Dox	1058776	1020515 to 1097037	Yes	****	<0.0001
**Figure 2H -** ***Alatina alata***					
**Time point = 0 mins**					
Venom Alone vs. 0.1 mM Dox	10445	-8328 to 29218	No	ns	0.4696
Venom Alone vs. 0.2 mM Dox	31544	12771 to 50317	Yes	***	0.0003
Venom Alone vs. 0.4 mM Dox	67183	48410 to 85956	Yes	****	<0.0001
Venom Alone vs. 2 mM Dox	119964	101191 to 138737	Yes	****	<0.0001
Venom Alone vs. 20 mM Dox	77658	58885 to 96431	Yes	****	<0.0001
**Time point = 15 mins**					
Venom Alone vs. 0.1 mM Dox	79486	60713 to 98259	Yes	****	<0.0001
Venom Alone vs. 0.2 mM Dox	161980	143207 to 180753	Yes	****	<0.0001
Venom Alone vs. 0.4 mM Dox	294105	275332 to 312878	Yes	****	<0.0001
Venom Alone vs. 2 mM Dox	323799	305026 to 342572	Yes	****	<0.0001
Venom Alone vs. 20 mM Dox	278858	260085 to 297631	Yes	****	<0.0001
**Time point = 30 mins**					
Venom Alone vs. 0.1 mM Dox	148285	129512 to 167058	Yes	****	<0.0001
Venom Alone vs. 0.2 mM Dox	297077	278304 to 315850	Yes	****	<0.0001
Venom Alone vs. 0.4 mM Dox	491873	473100 to 510645	Yes	****	<0.0001
Venom Alone vs. 2 mM Dox	522284	503511 to 541057	Yes	****	<0.0001
Venom Alone vs. 20 mM Dox	480786	462013 to 499559	Yes	****	<0.0001
**Time point = 45 mins**					
Venom Alone vs. 0.1 mM Dox	193776	175003 to 212549	Yes	****	<0.0001
Venom Alone vs. 0.2 mM Dox	386966	368193 to 405739	Yes	****	<0.0001
Venom Alone vs. 0.4 mM Dox	621942	603169 to 640715	Yes	****	<0.0001
Venom Alone vs. 2 mM Dox	651139	632367 to 669912	Yes	****	<0.0001
Venom Alone vs. 20 mM Dox	612138	593365 to 630910	Yes	****	<0.0001

**Time point = 60 mins**					
Venom Alone vs. 0.1 mM Dox	233638	214865 to 252411	Yes	****	<0.0001
Venom Alone vs. 0.2 mM Dox	457497	438724 to 476270	Yes	****	<0.0001
Venom Alone vs. 0.4 mM Dox	726756	707983 to 745529	Yes	****	<0.0001
Venom Alone vs. 2 mM Dox	754841	736068 to 773613	Yes	****	<0.0001
Venom Alone vs. 20 mM Dox	715099	696326 to 733871	Yes	****	<0.0001
**Figure 2I - Bee Venom PLA2**					
**Time point = 0 mins**					
**Dunnett's multiple comparisons test**	**Mean Diff.**	**95.00% CI of diff.**	**Below threshold?**	**Summary**	**Adjusted P Value**
Venom Alone vs. 0.01 mM CuGluc	90317	-369910 to 550544	No	ns	0.9816
Venom Alone vs. 0.1 mM CuGluc	198797	-261430 to 659024	No	ns	0.6953
Venom Alone vs. 1 mM CuGluc	835423	375196 to 1295650	Yes	****	<0.0001
Venom Alone vs. 10 mM CuGluc	2612552	2152325 to 3072779	Yes	****	<0.0001
Venom Alone vs. 100 mM CuGluc	3841454	3381227 to 4301681	Yes	****	<0.0001
**Time point = 15 mins**					
Venom Alone vs. 0.01 mM CuGluc	200281	-259946 to 660508	No	ns	0.6894
Venom Alone vs. 0.1 mM CuGluc	283875	-176352 to 744102	No	ns	0.3717
Venom Alone vs. 1 mM CuGluc	1157448	697221 to 1617675	Yes	****	<0.0001
Venom Alone vs. 10 mM CuGluc	5018171	4557944 to 5478398	Yes	****	<0.0001
Venom Alone vs. 100 mM CuGluc	7630510	7170283 to 8090737	Yes	****	<0.0001
**Time point = 30 mins**					
Venom Alone vs. 0.01 mM CuGluc	169909	-290318 to 630136	No	ns	0.8039
Venom Alone vs. 0.1 mM CuGluc	254408	-205819 to 714635	No	ns	0.4758
Venom Alone vs. 1 mM CuGluc	1098391	638164 to 1558618	Yes	****	<0.0001
Venom Alone vs. 10 mM CuGluc	4624512	4164285 to 5084739	Yes	****	<0.0001
Venom Alone vs. 100 mM CuGluc	7628078	7167851 to 8088305	Yes	****	<0.0001
**Time point = 45 mins**					
Venom Alone vs. 0.01 mM CuGluc	162855	-297372 to 623082	No	ns	0.828
Venom Alone vs. 0.1 mM CuGluc	250089	-210138 to 710316	No	ns	0.4921
Venom Alone vs. 1 mM CuGluc	1146193	685966 to 1606420	Yes	****	<0.0001
Venom Alone vs. 10 mM CuGluc	4726125	4265898 to 5186352	Yes	****	<0.0001
Venom Alone vs. 100 mM CuGluc	8170943	7710716 to 8631170	Yes	****	<0.0001
**Time point = 60 mins**					
Venom Alone vs. 0.01 mM CuGluc	158876	-301351 to 619103	No	ns	0.841
Venom Alone vs. 0.1 mM CuGluc	244853	-215374 to 705080	No	ns	0.5122
Venom Alone vs. 1 mM CuGluc	1139029	678802 to 1599256	Yes	****	<0.0001
Venom Alone vs. 10 mM CuGluc	4558887	4098660 to 5019114	Yes	****	<0.0001
Venom Alone vs. 100 mM CuGluc	8105651	7645424 to 8565878	Yes	****	<0.0001
**Figure 2J - *Naja kaouthia***					
**Time point = 0 mins**					
Venom Alone vs. 0.01 mM CuGluc	-91914	-345677 to 161849	No	ns	0.815
Venom Alone vs. 0.1 mM CuGluc	11612	-242151 to 265375	No	ns	>0.9999

Dunnett's multiple comparisons test	Mean Diff.	95.00% CI of diff.	Below threshold?	Summary	Adjusted P Value
Venom Alone vs. 1 mM CuGluc	134741	-119022 to 388504	No	ns	0.5141
Venom Alone vs. 10 mM CuGluc	518269	264505 to 772032	Yes	****	<0.0001
Venom Alone vs. 100 mM CuGluc	1066843	813080 to 1320606	Yes	****	<0.0001
**Time point = 15 mins**					
Venom Alone vs. 0.01 mM CuGluc	-80077	-333840 to 173686	No	ns	0.882
Venom Alone vs. 0.1 mM CuGluc	327456	73693 to 581219	Yes	**	0.0066
Venom Alone vs. 1 mM CuGluc	526535	272772 to 780298	Yes	****	<0.0001
Venom Alone vs. 10 mM CuGluc	3668219	3414456 to 3921982	Yes	****	<0.0001
Venom Alone vs. 100 mM CuGluc	6376139	6122375 to 6629902	Yes	****	<0.0001
**Time point = 30 mins**					
Venom Alone vs. 0.01 mM CuGluc	-133597	-387360 to 120166	No	ns	0.5221
Venom Alone vs. 0.1 mM CuGluc	267478	13715 to 521241	Yes	*	0.0353
Venom Alone vs. 1 mM CuGluc	436866	183103 to 690629	Yes	***	0.0002
Venom Alone vs. 10 mM CuGluc	3532207	3278444 to 3785970	Yes	****	<0.0001
Venom Alone vs. 100 mM CuGluc	7140952	6887188 to 7394715	Yes	****	<0.0001
**Time point = 45 mins**					
Venom Alone vs. 0.01 mM CuGluc	-155614	-409377 to 98149	No	ns	0.3772
Venom Alone vs. 0.1 mM CuGluc	291176	37413 to 544939	Yes	*	0.0188
Venom Alone vs. 1 mM CuGluc	465931	212168 to 719694	Yes	****	<0.0001
Venom Alone vs. 10 mM CuGluc	3576140	3322377 to 3829903	Yes	****	<0.0001
Venom Alone vs. 100 mM CuGluc	7667843	7414080 to 7921606	Yes	****	<0.0001
**Time point = 60 mins**					
Venom Alone vs. 0.01 mM CuGluc	-170191	-423954 to 83572	No	ns	0.2955
Venom Alone vs. 0.1 mM CuGluc	298122	44359 to 551885	Yes	*	0.0155
Venom Alone vs. 1 mM CuGluc	480722	226959 to 734485	Yes	****	<0.0001
Venom Alone vs. 10 mM CuGluc	3460434	3206671 to 3714197	Yes	****	<0.0001
Venom Alone vs. 100 mM CuGluc	7688159	7434396 to 7941923	Yes	****	<0.0001
**Figure 2K - *Daboia russelii***					
**Time point = 0 mins**					
Venom Alone vs. 0.01 mM CuGluc	85780	-122790 to 294350	No	ns	0.7324
Venom Alone vs. 0.1 mM CuGluc	74187	-134383 to 282757	No	ns	0.8251
Venom Alone vs. 1 mM CuGluc	48445	-160125 to 257015	No	ns	0.9628
Venom Alone vs. 10 mM CuGluc	261528	52958 to 470098	Yes	**	0.0087
Venom Alone vs. 100 mM CuGluc	533002	324432 to 741572	Yes	****	<0.0001
**Time point = 15 mins**					
**Dunnett's multiple comparisons test**	**Mean Diff.**	**95.00% CI of diff.**	**Below threshold?**	**Summary**	**Adjusted P Value**
Venom Alone vs. 0.01 mM CuGluc	117974	-90596 to 326544	No	ns	0.4539
Venom Alone vs. 0.1 mM CuGluc	139189	-69381 to 347759	No	ns	0.2999
Venom Alone vs. 1 mM CuGluc	224841	16271 to 433411	Yes	*	0.0302
Venom Alone vs. 10 mM CuGluc	1239999	1031429 to 1448569	Yes	****	<0.0001
Venom Alone vs. 100 mM CuGluc	2384676	2176106 to 2593246	Yes	****	<0.0001

**Time point = 30 mins**					
Venom Alone vs. 0.01 mM CuGluc	117889	-90681 to 326459	No	ns	0.4546
Venom Alone vs. 0.1 mM CuGluc	127124	-81446 to 335694	No	ns	0.3829
Venom Alone vs. 1 mM CuGluc	315115	106545 to 523685	Yes	**	0.0011
Venom Alone vs. 10 mM CuGluc	1955790	1747220 to 2164360	Yes	****	<0.0001
Venom Alone vs. 100 mM CuGluc	3767834	3559264 to 3976404	Yes	****	<0.0001
**Time point = 45 mins**					
Venom Alone vs. 0.01 mM CuGluc	107702	-100868 to 316272	No	ns	0.5402
Venom Alone vs. 0.1 mM CuGluc	105112	-103458 to 313682	No	ns	0.5628
Venom Alone vs. 1 mM CuGluc	346639	138069 to 555209	Yes	***	0.0003
Venom Alone vs. 10 mM CuGluc	2129618	1921048 to 2338188	Yes	****	<0.0001
Venom Alone vs. 100 mM CuGluc	4348752	4140182 to 4557322	Yes	****	<0.0001
**Time point = 60 mins**					
Venom Alone vs. 0.01 mM CuGluc	121012	-87558 to 329582	No	ns	0.4296
Venom Alone vs. 0.1 mM CuGluc	101850	-106720 to 310420	No	ns	0.5915
Venom Alone vs. 1 mM CuGluc	385180	176610 to 593750	Yes	****	<0.0001
Venom Alone vs. 10 mM CuGluc	2352256	2143686 to 2560826	Yes	****	<0.0001
Venom Alone vs. 100 mM CuGluc	5020240	4811670 to 5228810	Yes	****	<0.0001
**Figure 2L - *Alatina alata***					
**Time point = 0 mins**					
Venom Alone vs. 0.01 mM CuGluc	130923	-211809 to 473655	No	ns	0.7829
Venom Alone vs. 0.1 mM CuGluc	81433	-261299 to 424165	No	ns	0.9592
Venom Alone vs. 1 mM CuGluc	118703	-224029 to 461435	No	ns	0.8393
Venom Alone vs. 10 mM CuGluc	622121	279389 to 964853	Yes	****	<0.0001
Venom Alone vs. 100 mM CuGluc	1194519	851787 to 1537250	Yes	****	<0.0001
**Time point = 15 mins**					
Venom Alone vs. 0.01 mM CuGluc	146375	-196357 to 489107	No	ns	0.7041
Venom Alone vs. 0.1 mM CuGluc	19735	-322997 to 362467	No	ns	>0.9999
Venom Alone vs. 1 mM CuGluc	195855	-146877 to 538587	No	ns	0.4441
Venom Alone vs. 10 mM CuGluc	1062533	719801 to 1405265	Yes	****	<0.0001
**Dunnett's multiple comparisons test**	**Mean Diff.**	**95.00% CI of diff.**	**Below threshold?**	**Summary**	**Adjusted P Value**
Venom Alone vs. 100 mM CuGluc	2174148	1831416 to 2516879	Yes	****	<0.0001
**Time point = 30 mins**					
Venom Alone vs. 0.01 mM CuGluc	123779	-218953 to 466511	No	ns	0.8167
Venom Alone vs. 0.1 mM CuGluc	10234	-332498 to 352966	No	ns	>0.9999
Venom Alone vs. 1 mM CuGluc	243714	-99018 to 586446	No	ns	0.2457
Venom Alone vs. 10 mM CuGluc	1434967	1092235 to 1777699	Yes	****	<0.0001
Venom Alone vs. 100 mM CuGluc	2898596	2555864 to 3241328	Yes	****	<0.0001
**Time point = 45 mins**					
Venom Alone vs. 0.01 mM CuGluc	132252	-210480 to 474984	No	ns	0.7764
Venom Alone vs. 0.1 mM CuGluc	1845	-340887 to 344577	No	ns	>0.9999
Venom Alone vs. 1 mM CuGluc	279668	-63064 to 622400	No	ns	0.1454

Venom Alone vs. 10 mM CuGluc	1691286	1348554 to 2034018	Yes	****	<0.0001
Venom Alone vs. 100 mM CuGluc	3377291	3034559 to 3720023	Yes	****	<0.0001
**Time point = 60 mins**					
Venom Alone vs. 0.01 mM CuGluc	145207	-197525 to 487939	No	ns	0.7102
Venom Alone vs. 0.1 mM CuGluc	-10996	-353728 to 331736	No	ns	>0.9999
Venom Alone vs. 1 mM CuGluc	327646	-15086 to 670378	No	ns	0.0656
Venom Alone vs. 10 mM CuGluc	2025697	1682965 to 2368429	Yes	****	<0.0001
Venom Alone vs. 100 mM CuGluc	4058985	3716253 to 4401717	Yes	****	<0.0001
**Figure 2M - Bee Venom PLA2**					
**Time point = 0 mins**					
Venom Alone vs. 3 mM NALC	-240954	-506117 to 24209	No	ns	0.0868
Venom Alone vs. 6 mM NALC	-104572	-369735 to 160591	No	ns	0.7619
Venom Alone vs. 30 mM NALC	205349	-59814 to 470512	No	ns	0.1804
Venom Alone vs. 60 mM NALC	1350434	1085271 to 1615597	Yes	****	<0.0001
Venom Alone vs. 300 mM NALC	1280821	1015658 to 1545984	Yes	****	<0.0001
**Time point = 15 mins**					
Venom Alone vs. 3 mM NALC	-137038	-402201 to 128125	No	ns	0.5395
Venom Alone vs. 6 mM NALC	-39371	-304534 to 225792	No	ns	0.9947
Venom Alone vs. 30 mM NALC	369747	104584 to 634910	Yes	**	0.003
Venom Alone vs. 60 mM NALC	4609783	4344620 to 4874946	Yes	****	<0.0001
Venom Alone vs. 300 mM NALC	4523618	4258455 to 4788781	Yes	****	<0.0001
**Time point = 30 mins**					
Venom Alone vs. 3 mM NALC	-117138	-382301 to 148025	No	ns	0.6774
Venom Alone vs. 6 mM NALC	-49737	-314900 to 215426	No	ns	0.9849
Venom Alone vs. 30 mM NALC	214461	-50702 to 479624	No	ns	0.1511
Venom Alone vs. 60 mM NALC	5075060	4809897 to 5340223	Yes	****	<0.0001
Venom Alone vs. 300 mM NALC	4980458	4715295 to 5245621	Yes	****	<0.0001
**Time point = 45 mins**					
Venom Alone vs. 3 mM NALC	-93093	-358256 to 172070	No	ns	0.8322
Venom Alone vs. 6 mM NALC	-27937	-293100 to 237226	No	ns	0.9989
Venom Alone vs. 30 mM NALC	177129	-88034 to 442292	No	ns	0.299
Venom Alone vs. 60 mM NALC	5104722	4839559 to 5369885	Yes	****	<0.0001
Venom Alone vs. 300 mM NALC	5018358	4753195 to 5283521	Yes	****	<0.0001
**Time point = 60 mins**					
Venom Alone vs. 3 mM NALC	-79520	-344683 to 185643	No	ns	0.9013
Venom Alone vs. 6 mM NALC	-33488	-298651 to 231675	No	ns	0.9975
Venom Alone vs. 30 mM NALC	152536	-112627 to 417699	No	ns	0.4378
Venom Alone vs. 60 mM NALC	5068408	4803245 to 5333571	Yes	****	<0.0001
Venom Alone vs. 300 mM NALC	4978615	4713452 to 5243778	Yes	****	<0.0001
**Figure 2N - *Naja kaouthia***					
**Time point = 0 mins**					
Venom Alone vs. 3 mM NALC	-15961	-65341 to 33419	No	ns	0.872
Venom Alone vs. 6 mM NALC	-11655	-61035 to 37725	No	ns	0.9603

Venom Alone vs. 30 mM NALC	-24565	-73945 to 24815	No	ns	0.5747
Venom Alone vs. 60 mM NALC	100014	50634 to 149394	Yes	****	<0.0001
Venom Alone vs. 300 mM NALC	99517	50137 to 148897	Yes	****	<0.0001
**Time point = 15 mins**					
Venom Alone vs. 3 mM NALC	-110131	-159511 to -60751	Yes	****	<0.0001
Venom Alone vs. 6 mM NALC	-117100	-166480 to -67720	Yes	****	<0.0001
Venom Alone vs. 30 mM NALC	-47681	-97061 to 1699	No	ns	0.0619
Venom Alone vs. 60 mM NALC	542383	493003 to 591763	Yes	****	<0.0001
Venom Alone vs. 300 mM NALC	565278	515898 to 614658	Yes	****	<0.0001
**Time point = 30 mins**					
Venom Alone vs. 3 mM NALC	-56900	-106280 to -7520	Yes	*	0.0182
Venom Alone vs. 6 mM NALC	-68424	-117804 to -19044	Yes	**	0.0032
Venom Alone vs. 30 mM NALC	14240	-35140 to 63620	No	ns	0.9143
Venom Alone vs. 60 mM NALC	738972	689592 to 788352	Yes	****	<0.0001
Venom Alone vs. 300 mM NALC	759289	709909 to 808669	Yes	****	<0.0001
**Time point = 45 mins**					
Venom Alone vs. 3 mM NALC	-36576	-85956 to 12804	No	ns	0.2133
**Dunnett's multiple comparisons test**	**Mean Diff.**	**95.00% CI of diff.**	**Below threshold?**	**Summary**	**Adjusted P Value**
Venom Alone vs. 6 mM NALC	-49146	-98526 to 234.0	No	ns	0.0515
Venom Alone vs. 30 mM NALC	25704	-23676 to 75084	No	ns	0.5327
Venom Alone vs. 60 mM NALC	838879	789499 to 888259	Yes	****	<0.0001
Venom Alone vs. 300 mM NALC	857878	808498 to 907258	Yes	****	<0.0001
**Time point = 60 mins**					
Venom Alone vs. 3 mM NALC	-26991	-76371 to 22389	No	ns	0.4866
Venom Alone vs. 6 mM NALC	-41898	-91278 to 7482	No	ns	0.122
Venom Alone vs. 30 mM NALC	20811	-28569 to 70191	No	ns	0.7143
Venom Alone vs. 60 mM NALC	871345	821965 to 920725	Yes	****	<0.0001
Venom Alone vs. 300 mM NALC	889905	840525 to 939285	Yes	****	<0.0001
**Figure 2O - *Daboia russelii***					
**Time point = 0 mins**					
Venom Alone vs. 3 mM NALC	-19178	-85881 to 47525	No	ns	0.9152
Venom Alone vs. 6 mM NALC	-15886	-82589 to 50817	No	ns	0.9589
Venom Alone vs. 30 mM NALC	-48977	-115680 to 17726	No	ns	0.2201
Venom Alone vs. 60 mM NALC	415810	349107 to 482513	Yes	****	<0.0001
Venom Alone vs. 300 mM NALC	459611	392908 to 526314	Yes	****	<0.0001
**Time point = 15 mins**					
Venom Alone vs. 3 mM NALC	-36021	-102724 to 30682	No	ns	0.4981
Venom Alone vs. 6 mM NALC	-36642	-103345 to 30061	No	ns	0.4818
Venom Alone vs. 30 mM NALC	-28489	-95192 to 38214	No	ns	0.7041
Venom Alone vs. 60 mM NALC	887288	820585 to 953991	Yes	****	<0.0001
Venom Alone vs. 300 mM NALC	952051	885348 to 1018754	Yes	****	<0.0001
**Time point = 30 mins**					

Venom Alone vs. 3 mM NALC	-36686	-103389 to 30017	No	ns	0.4807
Venom Alone vs. 6 mM NALC	-39849	-106552 to 26854	No	ns	0.4018
Venom Alone vs. 30 mM NALC	-31586	-98289 to 35117	No	ns	0.6188
Venom Alone vs. 60 mM NALC	963339	896636 to 1030042	Yes	****	<0.0001
Venom Alone vs. 300 mM NALC	1034823	968120 to 1101526	Yes	****	<0.0001
**Time point = 45 mins**					
Venom Alone vs. 3 mM NALC	-39011	-105714 to 27692	No	ns	0.422
Venom Alone vs. 6 mM NALC	-42143	-108846 to 24560	No	ns	0.3494
Venom Alone vs. 30 mM NALC	-34204	-100907 to 32499	No	ns	0.5467
Venom Alone vs. 60 mM NALC	973294	906591 to 1039997	Yes	****	<0.0001
Venom Alone vs. 300 mM NALC	1050255	983552 to 1116958	Yes	****	<0.0001
**Time point = 60 mins**					
**Dunnett's multiple comparisons test**	**Mean Diff.**	**95.00% CI of diff.**	**Below threshold?**	**Summary**	**Adjusted P Value**
Venom Alone vs. 3 mM NALC	-36683	-103386 to 30020	No	ns	0.4807
Venom Alone vs. 6 mM NALC	-43554	-110257 to 23149	No	ns	0.3193
Venom Alone vs. 30 mM NALC	-36112	-102815 to 30591	No	ns	0.4957
Venom Alone vs. 60 mM NALC	977468	910765 to 1044171	Yes	****	<0.0001
Venom Alone vs. 300 mM NALC	1058835	992132 to 1125538	Yes	****	<0.0001
**Figure 2P -** ***Alatina alata***					
**Time point = 0 mins**					
Venom Alone vs. 3 mM NALC	-5171	-25177 to 14835	No	ns	0.9432
Venom Alone vs. 6 mM NALC	2987	-17019 to 22993	No	ns	0.9946
Venom Alone vs. 30 mM NALC	12367	-7639 to 32373	No	ns	0.3697
Venom Alone vs. 60 mM NALC	24518	4512 to 44524	Yes	*	0.0107
Venom Alone vs. 300 mM NALC	25407	5401 to 45413	Yes	**	0.0077
**Time point = 15 mins**					
Venom Alone vs. 3 mM NALC	-5624	-25630 to 14383	No	ns	0.9219
Venom Alone vs. 6 mM NALC	10557	-9449 to 30563	No	ns	0.5199
Venom Alone vs. 30 mM NALC	48851	28845 to 68857	Yes	****	<0.0001
Venom Alone vs. 60 mM NALC	197473	177467 to 217480	Yes	****	<0.0001
Venom Alone vs. 300 mM NALC	199475	179469 to 219481	Yes	****	<0.0001
**Time point = 30 mins**					
Venom Alone vs. 3 mM NALC	-6204	-26210 to 13802	No	ns	0.8889
Venom Alone vs. 6 mM NALC	19182	-824.3 to 39188	No	ns	0.0645
Venom Alone vs. 30 mM NALC	87167	67160 to 107173	Yes	****	<0.0001
Venom Alone vs. 60 mM NALC	370798	350792 to 390804	Yes	****	<0.0001
Venom Alone vs. 300 mM NALC	370810	350804 to 390816	Yes	****	<0.0001
**Time point = 45 mins**					
Venom Alone vs. 3 mM NALC	-5837	-25843 to 14169	No	ns	0.9106
Venom Alone vs. 6 mM NALC	25315	5309 to 45322	Yes	**	0.008
Venom Alone vs. 30 mM NALC	107950	87943 to 127956	Yes	****	<0.0001
Venom Alone vs. 60 mM NALC	492804	472798 to 512810	Yes	****	<0.0001

Venom Alone vs. 300 mM NALC	493126	473120 to 513133	Yes	****	<0.0001
**Time point = 60 mins**					
Venom Alone vs. 3 mM NALC	-8018	-28024 to 11988	No	ns	0.7508
Venom Alone vs. 6 mM NALC	29801	9794 to 49807	Yes	**	0.0014
Venom Alone vs. 30 mM NALC	120376	100369 to 140382	Yes	****	<0.0001
Venom Alone vs. 60 mM NALC	604938	584931 to 624944	Yes	****	<0.0001
Venom Alone vs. 300 mM NALC	604661	584654 to 624667	Yes	****	<0.0001

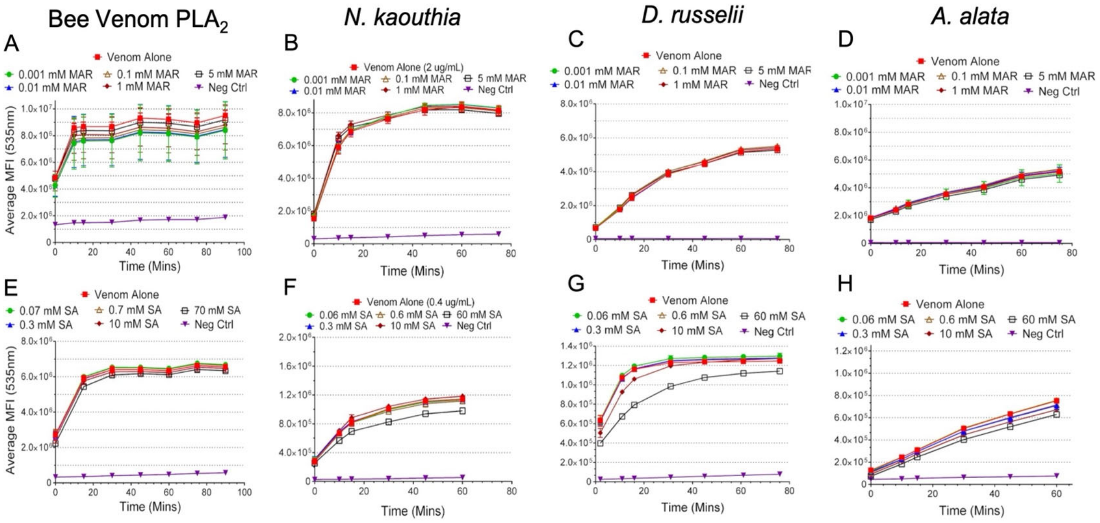

Figure S1. Marimastat and sodium aurothiomalate inhibition of PLA_2 activity. Inhibition of bee venom PLA_2 (0.4 U/mL), *N. kaouthia* (0.4 μg/mL or 2 μg/mL), *D. russelii* (200 μg/mL), or *A. alata* (200 μg/mL) venom by (A-D) MAR, marimastat and (E-H) SA, sodium aurothiomalate. Each curve represents the mean of three measurements and the error bars represent standard error of the mean (SEM).
